# *Post-hoc* Labeling of Arbitrary M/EEG Recordings for Data-Efficient Evaluation of Neural Decoding Methods

**DOI:** 10.3389/fninf.2019.00055

**Published:** 2019-08-02

**Authors:** Sebastián Castaño-Candamil, Andreas Meinel, Michael Tangermann

**Affiliations:** ^1^Brain State Decoding Lab, Department of Computer Science and BrainLinks-BrainTools Cluster of Excellence, University of Freiburg, Freiburg, Germany; ^2^Autonomous Intelligent Systems, Department of Computer Science, University of Freiburg, Freiburg, Germany

**Keywords:** neural decoding, M/EEG labeling, data-driven neural decoding, brain computer interfaces, band-power decoding

## Abstract

Many cognitive, sensory and motor processes have correlates in oscillatory neural source activity, which is embedded as a subspace in the recorded brain signals. Decoding such processes from noisy magnetoencephalogram/electroencephalogram (M/EEG) signals usually requires data-driven analysis methods. The objective evaluation of such decoding algorithms on experimental raw signals, however, is a challenge: the amount of available M/EEG data typically is limited, labels can be unreliable, and raw signals often are contaminated with artifacts. To overcome some of these problems, simulation frameworks have been introduced which support the development of data-driven decoding algorithms and their benchmarking. For generating artificial brain signals, however, most of the existing frameworks make strong and partially unrealistic assumptions about brain activity. This limits the generalization of results observed in the simulation to real-world scenarios. In the present contribution, we show how to overcome several shortcomings of existing simulation frameworks. We propose a versatile alternative, which allows for an objective evaluation and benchmarking of novel decoding algorithms using real neural signals. It allows to generate comparatively large datasets with labels being deterministically recoverable from the arbitrary M/EEG recordings. A novel idea to generate these labels is central to this framework: we determine a subspace of the true M/EEG recordings and utilize it to derive novel labels. These labels contain realistic information about the oscillatory activity of some underlying neural sources. For two categories of subspace-defining methods, we showcase how such labels can be obtained—either by an exclusively data-driven approach (independent component analysis—ICA), or by a method exploiting additional anatomical constraints (minimum norm estimates—MNE). We term our framework *post-hoc labeling* of M/EEG recordings. To support the adoption of the framework by practitioners, we have exemplified its use by benchmarking three standard decoding methods—i.e., common spatial patterns (CSP), source power-comodulation (SPoC), and convolutional neural networks (ConvNets)—wrt. Varied dataset sizes, label noise, and label variability. Source code and data are made available to the reader for facilitating the application of our *post-hoc* labeling framework.

## 1. Introduction

Brain oscillatory phenomena measured with non-invasive imaging techniques, such as magneto- or electroencephalography (M/EEG), contain information about underlying neural processes. The possibility to describe these is a prerequisite to answer questions appearing in clinical contexts, as well as in basic neuroscience research. Examples are the monitoring of rehabilitation progress, the characterization of neurological and neuropsychiatric disorders (Herrmann and Demiralp, 2005), the investigation of memory processes (Klimesch et al., 2007), motor performance (Tangermann et al., 2015; Meinel et al., 2016), and visual perception (Marshall et al., 2018).

### 1.1. Benchmarking and Validation of Data-Driven Neural Decoding Algorithms

Since the rise of brain-computer interface (BCI) systems, great effort has been put into developing novel techniques for decoding neural sources from noisy M/EEG recordings using linear and nonlinear methods, both for classification and regression tasks (Lotte et al., 2018). For the development, validation, and benchmarking of such neural decoding algorithms, it is desirable to have multichannel datasets with large amounts of labeled data available. In the literature, two types of frameworks prevail. First, frameworks making use of real M/EEG recordings acquired during experimental sessions, and second, those using synthetically generated pseudo-M/EEG signals. Each comes with advantages and shortcomings, as explained below.

#### 1.1.1. Real M/EEG Recordings

Using recordings of M/EEG data has the great advantage that their dynamics, the signal-to-noise ratio between oscillatory sources of interest and undesired background activity as well as any non-stationary behavior over time are provided naturally, such that they do not need to be set by the experimenter. Efforts have been made to provide benchmarking platforms, e.g., Jayaram and Barachant (2018) which contains real M/EEG data that has been recorded under specific experimental paradigms. However, the limitations of using such paradigm-specific M/EEG data remains.

##### 1.1.1.1. Small Datasets

The amount of labeled real M/EEG data acquired in a single experimental session maximally lasts a couple of hours. This limited dataset size is rendered even smaller by subsequent data preprocessing steps, i.e., data segmentation, removal of inter-trial pauses and rejection of artifactual segments. Bigger datasets may be obtained by applying transfer learning techniques, with the aim of merging inter-subject and inter-session data (Krauledat et al., 2008). However, this comes with its own substantial challenges and is subject to active research (Jayaram et al., 2016; Lotte et al., 2018). Overall, the relative small dataset size is a clear drawback of using real M/EEG data for the benchmarking of algorithms.

##### 1.1.1.2. Label Noise

In some experimental setups, M/EEG recordings are governed by a varying but known experimental parameter—such as the intensity of an external stimulus (Dähne et al., 2014b)—this parameter can be used as a target variable ***z***, which serves as epoch-wise labels to support the supervised decoding of correlated oscillatory M/EEG activity. Unfortunately, the situation is more challenging, if an M/EEG correlate of open behavior or even an imagery task shall be decoded: a *reliable* behavioral surrogate is lacking, and the precise registration of open behavior may also be difficult, thus, the investigator may end up with a noisy estimate. This label noise can have many different origins: subjects may be unable to execute the task with a required timing, they may not follow the experimental instructions consistently, may change their mental strategies to solve a problem, or display varying levels of engagement over time. Compared to clean labels ***z***, noisy label information is known to decrease the performance of decoding algorithms (Castaño-Candamil et al., 2015b). A number of decoding tasks, like the estimation of the motor tasks in imagery experiments (Höhne et al., 2014) in BCI, the prediction of hand motor performance (Meinel et al., 2016), or attention decoding (Martel et al., 2014), are considered very challenging, with label noise as a substantial part of the problem. As the experimenter typically neither knows the level of label noise contained in ***z*** nor can control it, behavioral experiments deliver suboptimal data for the benchmarking of decoding algorithms.

##### 1.1.1.3. Task-Specific

Last but not least, the use of real M/EEG data for benchmarking comes with the drawback that switching between decoding approaches, e.g., classification and regression, may require to redesign M/EEG experiments and run them again to collect the necessary novel label types.

#### 1.1.2. Synthetic Pseudo-M/EEG Signals

Some shortcomings of real data can be mitigated with synthetically generated M/EEG signals (Krol et al., 2018), which are utilized preferably in the fields of brain mapping and connectivity analysis (Castaño-Candamil et al., 2015a; Haufe and Ewald, 2016). Here, the assumption of a linear mapping from the neural source space to the M/EEG sensor space allows to simulate a neural target source, whose activity overlaps with measurement noise and task-irrelevant brain activity termed *background sources*. Special attention is dedicated to the modeling of sources, such that they match naturally occurring frequency spectra, e.g., reproducing a 1/*f*-shaped frequency spectrum and a narrow-band oscillatory target source. However, since these simulations are based purely on synthetic data, they need to make strong assumptions about brain dynamics.

##### 1.1.2.1. Assumptions About Neural Dynamics

The assumptions made are expressed by the choices of, e.g., the power ratio between target- and background sources, the noise level on the sensor space, and the time series of the sources. Synthetic datasets typically disregard more complex dynamics, which are present in real datasets and pose substantial challenges for decoding methods. While being sufficient for proof-of-concept purposes (Dähne et al., 2014b; Lindgren et al., 2018), these purely synthetic datasets lack a sufficient level of realism to allow for generalizing simulated performance estimates to real-world scenarios.

##### 1.1.2.2. Physiologically- and Functionally-Motivated Models

Simulated M/EEG time series are also used intensively in the field of computational neuroscience. Here, physiologically motivated linear and nonlinear stochastic systems are utilized to describe e.g., the dynamics of Alzheimer's disease, epilepsy, or sleeping disorders (Robinson et al., 2002; Kim et al., 2007) on the level of small networks and M/EEG. The complexity of such methods span from linear univariate, to highly detailed multivariate models motivated by complex functional and physiological constraints. Univariate linear models disregard the notion of spatiality inherent to M/EEG recordings and resemble simple oscillators. Multivariate models—at the other end of the complexity spectrum—account for highly specific networks and dynamics in the brain, but require control over a large number of parameters (Breakspear et al., 2010). To determine them based on data is difficult and may succeed only when very large data collections are accessible. In contrast to the physiologically motivated systems, purely data-driven approaches using recurrent neural networks methods (Forney et al., 2015) have been explored. These approaches are capable of generating a single channel of realistic, albeit artificial, M/EEG time-series but also disregard the spatial notion present in M/EEG recordings.

### 1.2. *Post-hoc* Labeling of Paradigm-Agnostic M/EEG Recordings

Motivated by the shortcomings of using real M/EEG recordings (few data and noisy labels) as well as of synthetically generated datasets (questionable assumptions about neural dynamics and noise), we propose a novel generation framework for labeled datasets. It is based on *post-hoc* labeling of pre-recorded real M/EEG signals, that generates novel labels using unsupervised subspace projection methods. As the original labels of the dataset are discarded, the framework is agnostic wrt. the original paradigm under which the M/EEG signals had been recorded, and to its original trial structure.

As a result, with our framework we aim at obtaining datasets with the following properties:

- Real neural dynamics, as contained in the M/EEG signals;
- highly efficient use of real M/EEG data (thus potentially yielding larger datasets), and
- labels deterministically recoverable from the available data, i.e., free of noise.

Our proposed framework is compatible with existing benchmarking frameworks, for instance the Mother of All BCI Benchmarks (MOABB) introduced in Jayaram and Barachant (2018).

## 2. Methods

### 2.1. Generative Model of Brain Activity

Neural activity recorded by M/EEG can be represented by means of a linear forward model (Baillet et al., 2001; Grech et al., 2008):

(1) $$\begin{array}{l} X=AS+E, \end{array}$$

where $X\;\in C\subseteq {\mathbb{R}}^{N_{c}\times N_{t}}$ is a multivariate signal in the channel space C describing M/EEG data measured by *N*_*c*_ M/EEG channels at *N*_*t*_ discrete time samples, $S\;\in S\subseteq {\mathbb{R}}^{N_{s}\times N_{t}}$ describes the time course of *N*_*s*_ neural sources in the source space S with covariance matrix $Q\;\in \;{\mathbb{R}}^{N_{s}\times N_{s}}$; and matrix $A\;\in \;{\mathbb{R}}^{N_{c}\times N_{s}}$ describes the linear projection S→C of the sources onto the sensor space, where the columns of ***A***, $a\;\in \;{\mathbb{R}}^{N_{c}}$, are referred to as *spatial patterns*. Furthermore, the matrix ***E*** contains i.i.d. Gaussian noise with zero mean and a covariance matrix $Q_{\epsilon }\;\in \;{\mathbb{R}}^{N_{c}\times N_{c}}$.

Under this representation, it is widely accepted that surrogates of a wide range of cognitive processes can be decoded from the power of narrowband frequency oscillatory sources in ***S*** (Dähne et al., 2014b; Horschig et al., 2014). We will represent such a surrogate by the row vector $s_{z}^{\text{T}}\;\in \;{\mathbb{R}}^{N_{t}}$ of ***S***, whereas its envelope—the power of the source—will be denoted as $z\;\in \;{\mathbb{R}}^{N_{t}}$ and termed *target variable*. It delivers the labels and, consequently, represents the variable that is to be decoded for unseen data.

### 2.2. *Post-hoc* Labeling of Paradigm-Agnostic M/EEG Recordings

Our novel framework refrains from making (potentially problematic) assumptions about the dynamics of neural activity or about the signal-to-noise ratio between an oscillatory source of interest and background sources. The framework relies upon an unsupervised projection of an arbitrary M/EEG dataset ***X*** onto a source space S by means of a function f:C→S.

Assuming we can find such a function which decomposes the M/EEG signals into *reasonable* sources (the next paragraphs will deal with this), we also propose that any source in S could be selected to serve as the target source ***s***_*z*_ and that the oscillatory power of this source can be used to provide the labels ***z*** for the purpose of benchmarking arbitrary decoding methods.

#### 2.2.1. Determining *f* as a Linear Projection Function

We propose two alternative strategies to choose function ***f***: the first one uses an anatomically constrained source space $S_{a}\subseteq S$ while the second strategy defines the source space $S_{d}\subseteq S$ in a purely data-driven manner.

##### 2.2.1.1. Anatomically Constrained Source Space $S_{a}$

If an anatomically motivated head model ***A***, potentially containing a very large number of sources, is available (Hallez et al., 2007), then ***f*** can be selected such that ***X*** is projected onto an anatomically constrained version of the source space, $S_{a}$. To this end, a source reconstruction method may be used. Specifically, the maximum a-posteriori estimate of $S\in S_{a}$ can be found as the minimizer of the following cost function (Grech et al., 2008; Castaño-Candamil et al., 2015a):

(2) $$\begin{array}{l} \underset{S}{\mathrm{argmin}}\left\{||X-AS|{|}_{Q}^{2}+\lambda \Theta \left(S\right)\right\}. \end{array}$$

Here, ||**·**||_**Q**_ϵ__ is the matrix norm of the argument wrt. the covariance matrix ***Q***_ϵ_, and λ∈ℝ^+^ is a regularization constant. The penalty term Θ(**S**):**S** ↦ ℝ^+^ can be utilized to formalize arbitrary constraints imposed upon the neural source activity. Many different algorithms, each with specific choices for Θ(**S**) and ***Q***_ϵ_, have been introduced (Grech et al., 2008), each of them representing different priors about the expected characteristics of sources. For the sake of simplicity and assuming stationarity wrt. ***Q*** and ***Q***_ϵ_, we have chosen $\Theta \left(S\right)=||S|{|}_{2}^{2}$ and ***Q***_ϵ_ = ***I***_***N***_*c*__, where $I_{N_{c}}\in {\mathbb{R}}^{N_{c}\times N_{c}}$ is an identity matrix. This approach is commonly termed ℓ_2_-norm regularization (Ng, 2004), also known as minimum norm estimate (MNE) (Pascual-Marqui, 1999; Grech et al., 2008). For this choice of Θ, it can be shown that the optimal solution for expression 2 is given conveniently by

(3) $$\begin{array}{l} S=A^{\text{T}}{\left(I_{N_{c}}+\lambda AA^{\text{T}}\right)}^{-1}X \end{array}$$

where hyperparameter λ is determined, in our case, using the generalized crossvalidation procedure, as described in Grech et al. (2008).

Please note that the proposed *post-hoc* labeling framework is not limited to using MNE, and therefore, the assumption about stationary dynamics made by MNE is an external factor and is not intrinsically embedded in the proposed framework. If non-stationary dynamics in the underlying sources shall be taken into account, more complex mapping methods, as time-frequency mixed norm estimates (TF-MxNE) (Gramfort et al., 2013b) or spatio-temporal unifying tomography (STOUT) (Castaño-Candamil et al., 2015a) can be used to obtain ***S*** in $S_{a}$.

##### 2.2.1.2. Data-Driven Source Space $S_{d}$

Choosing a data-driven approach, a set of underlying target sources can be estimated from ***X*** using standard unsupervised linear decomposition methods such as PCA, ICA, factor analysis, among others. In the following, we use the fastICA algorithm (Hyvärinen and Oja, 2000), which—among the different blind source separation methods—has been widely employed for the analysis of neural data (Makeig et al., 1996; Vigário et al., 2000; Delorme and Makeig, 2004). For this choice, the function ***f*** is defined as ***S*** = ***f***(***X***) = **Φ*X***, where $\Phi \in {\mathbb{R}}^{N_{s}\times N_{c}}$ is a matrix spanning $S_{d}$, a space of maximally independent components. Independence is achieved by maximizing non-Gaussianity of the sources $S\in S_{d}$. Please note again, that the proposed dataset generation framework is not dependent on this specific choice of fastICA, and therefore, other blind source separation methods (including adaptive approaches, capable of dealing with non-stationary dynamics), can be used for estimating ***S*** in $S_{d}$.

#### 2.2.2. Extraction of Target Variable *z*

Once a set of sources has been determined by the two approaches mentioned above (or any analog thereof), a target source ***s***_*z*_ is to be selected. The choice could be guided by a prior about the benchmarking problem (e.g., strong components only, or components that stem from a brain region known to be involved in a certain experimental task) or simply by random selection. Similarly, a arbitrary combination of sources could also be selected as target source. In this regard, our *post-hoc* labeling framework offers absolute flexibility regarding the criteria used for obtaining the target source. After it has been selected, the labels ***z*** are computed in the following three-step procedure:

1. Since it is expected that the target source provides surrogate information about a cognitive process by means of its power in a narrow frequency band (Horschig et al., 2014), ***X*** is filtered with a bandpass filter to reflect this assumption and then projected onto S, where a target source ***s***_*z*_ is selected.
2. The band power envelope of the selected source is determined by computing, e.g., the magnitude of its Hilbert transform
  (4) $$\begin{array}{l} z=|ℋ\left\{s_{z}\right\}| \end{array}$$
3. If desired, the data and the labels can be segmented into epochs. It is important to remark that this step depends on the algorithm which shall be benchmarked later on.

**Figure 1:**
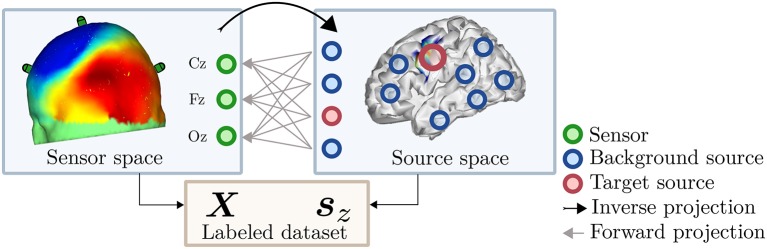
Illustration of the *post-hoc* labeling dataset generation framework. M/EEG recordings in the sensor space **(Left)** are represented by a scalp map. The information contained in these signals is mapped to the source space **(Right)** by a so-called inverse projection. This projection is the key ingredient for our *post-hoc* labeling and can be performed either via source reconstruction techniques or by unsupervised decomposition methods.

The dataset resulting from these steps consists of real EEG recordings ***X*** and a noiseless continuous variable ***z*** containing the corresponding target labels. Using ***X*** and ***z***, any *arbitrary* supervised decoding algorithm can be benchmarked and validated. Furthermore, the proposed formulation may be extended to obtain *discrete* labels ***y*** by assigning a class label depending on percentile memberships of individual labels *z*_*i*_, thus extending the applicability of our proposed framework to classification tasks. Figure 1 illustrates the general idea of the proposed *post-hoc* labeling framework for datasets generation.

## 3. Experimental Setup

### 3.1. Decoding Methods

Among many different neural decoding methods found in the literature, linear subspace decomposition methods come with the advantage of computational simplicity and offer interpretability of the decoded information (Haufe et al., 2014b). Besides extracting label-informative oscillatory components, these approaches are often put to work for dimensionality reduction before applying more advanced processing methods. The pioneering work on joint covariance diagonalization presented by Fukunaga (2013) and reformulated by de Cheveigné and Parra (2014) serves as a generalized foundation for popular supervised linear subspace decomposition algorithms. One representative is the common spatial patterns (CSP) algorithm, suitable for the classification of oscillatory processes related to motor execution, motor imagery, and attempted motor execution tasks (Koles et al., 1990; Lemm et al., 2005). The relevance of CSP is not only indicated by its extensive use (Tangermann et al., 2012), but also by the plethora of derivatives that have been introduced after its original presentation, like finite impulse response CSP (FIR-CSP) (Higashi and Tanaka, 2013), sub-band CSP (SBCSP) (Novi et al., 2007), filter-bank CSP (FBCSP) (Ang et al., 2008), spectrally weighted-CSP (SPEC-CSP) (Tomioka et al., 2006), among others. While CSP is a supervised algorithm preferred for classification problems, the source power comodulation algorithm SPoC (Dähne et al., 2014b), together with its extensions canonical SPoC (cSPoC) (Dähne et al., 2014c) and multimodal SPoC (mSPoC) (Dähne et al., 2014a), lends itself to solve supervised linear regression tasks (Meinel et al., 2016). Unsupervised linear neural decoding methods are also extremely popular: After principal component analysis (PCA), the most widely applied class of algorithms is probably the family of independent component analysis (ICA) methods, which realize blind source separation (Makeig et al., 1996). Last but not least, in the context of unsupervised extraction of specific oscillatory components, the spatio-spectral decomposition (SSD) approach introduced by Haufe et al. (2014a) deserves to be mentioned.

In addition to these linear subspace methods, nonlinear decoding methods have been introduced. A recent example are convolutional neural networks (ConvNets) used for, e.g., the classification of motor tasks (Schirrmeister et al., 2017), visually evoked potentials (Lawhern et al., 2016), error-related negativity responses, movement-related cortical potentials, and sensory motor rhythms. Further decoding approaches which make use of various machine learning models have been described in the literature (see the review provided in Schirrmeister et al., 2017; Lotte et al., 2018).

We implemented three different decoding methods to exemplify the application of a our *post-hoc*-labeling framework. Specifically, we will report on two classification tasks (using CSP and ConvNets for a two- and three class classification problem, respectively) and SPoC for a regression task, covering a wide range of popular decoding algorithms. We will benchmark these methods wrt. several dataset-inherent parameters, i.e., dataset size, label noise level, and variance of the target label ***z***. The performance of these decoding methods is evaluated using arbitrarily selected accuracy metrics for the sake of illustration, i.e., AUC, classification performance, or linear correlation coefficient.

#### 3.1.1. Common Spatial Patterns (CSP)

The CSP algorithm is an established supervised method in the BCI community, used in classification tasks for constructing a set of *N*_*c*_ spatial filters $W_{CSP}\in {\mathbb{R}}^{N_{c}\times N_{c}}$ that optimally discriminates epochs of two classes characterized by differing band-power features, where the labels are defined as ***y***∈{1, 2}, corresponding to a discretization of the continuous label ***z***.

Assuming that M/EEG data ***X*** have been bandpass filtered to the frequency band of interest and segmented into a set of *N* epochs, where ***X***(*e*) represent the *e*-th epoch of the M/EEG data, the CSP objective function is mathematically formalized as

(5) $$\begin{array}{l} \underset{W_{CSP}}{\mathrm{argmax}}\frac{W_{CSP}^{\text{T}}\left(C_{1}-C_{2}\right)W_{CSP}}{W_{CSP}^{\text{T}}CW_{CSP}}. \end{array}$$

with the spatial covariance matrices of classes one and two defined as $C_{1,2}=N_{1,2}^{-1}\overset{N}{\underset{e}{\sum }}X_{1,2}\left(e\right)X_{1,2}{\left(e\right)}^{\text{T}}$ and with ***C*** being the pooled spatial covariance matrix. It can be shown that a solution to the CSP optimization problem can be found by solving the generalized eigenvalue problem

(6) $$\begin{array}{l} W_{CSP}^{\text{T}}\left(C_{1}-C_{2}\right)=\Lambda W_{CSP}^{\text{T}}C \end{array}$$

with ***W***_*CSP*_ being a matrix containing (column-wise) the eigenvectors (i.e., spatial filters) which are related to the eigenvalues provided by the entries of the main diagonal of $\Lambda \in {\mathbb{R}}^{N_{f}\times N_{f}}$.

For our tests, we reduced the full filter matrix to ${\hat{W}}_{CSP}\in {\mathbb{R}}^{Nc\times 2}$. Thus, it contains two eigenvectors only, one corresponding to the largest and one to the smallest eigenvalues in **Λ**, representing each class, respectively. The selection of the number of CSP filters is an important hyperparameter for obtaining an optimal decoding performance. However, according to Blankertz et al. (2011), a good rule of thumb indicates that between 2 and 8 filters are likely to deliver a good performance.

Note that a spatial filters derived by CSP does not deliver an estimate of the target variable $\hat{z}\left(e\right)$ yet. To derive estimates of the target variable, we thus trained a regularized LDA (rLDA) classifier on the power features (delivered by $\Theta_{x}\left(e\right)=\mathrm{var}\left[\hat{s}\right]\left(e\right)$) of the spatially filtered data $\hat{s}\left(e\right)={\hat{W}}_{CSP}^{⊤}X\left(e\right)$ (Blankertz et al., 2011).

The results reported here were computed using the CSP implementation provided in the MNE toolbox by Gramfort et al. (2013a, 2014).

##### 3.1.1.1. Decoding Accuracy

Depending on the application, an arbitrary metric can be used, which matches well with the given decoding method, e.g., classification accuracy, Type I/II errors, etc. To characterize CSP performance we chose the area ander the ROC curve (AUC).

#### 3.1.2. Source Power Comodulation (SPoC)

Analogously to CSP, the multivariate neural decoding method SPoC (Dähne et al., 2014b) utilizes a supervised regression approach in order to estimate a spatial filter $w_{SPoC}\in {\mathbb{R}}^{N_{c}}$, onto which ***X*** will be linearly projected to extract the underlying *continuous* target source ***z***.

Specifically, a SPoC spatial filter $w_{SPoC}\in {\mathbb{R}}^{N_{c}}$ is optimized such that the power of a projected epoch, $\Theta_{x}\left(e\right)=\mathrm{var}\left[\hat{s}\right]\left(e\right)$, maximally covaries with the target variable ***z***(*e*):

(7) $$\begin{array}{l} \underset{w}{\mathrm{argmax}}\left\{\text{cov}\left[\Theta_{x}\left(e\right),z\left(e\right)\right]\right\}\forall e \end{array}$$

It can be shown that solving this optimization problem is equivalent to solving the generalized eigenvalue problem

(8) $$\begin{array}{l} C_{z}w_{SPoC}=\lambda Cw_{SPoC} \end{array}$$

where $C=N^{-1}\overset{N}{\underset{e}{\sum }}X\left(e\right)X{\left(e\right)}^{\text{T}}$ is the spatial covariance matrix of ***X*** and $C_{z}=N^{-1}\overset{N}{\underset{e}{\sum }}z\left(e\right)X\left(e\right)X{\left(e\right)}^{\text{T}}$ is the epoch-wise ***z***-weighted spatial covariance matrix.

Given a spatial filter ***w***_*SPoC*_, the target variable ***z*** can subsequently be estimated as $\hat{z}$ for each single epoch of unseen test data ***X***_*te*_ via $\hat{z}\left(e\right)=\mathrm{var}\left[w_{SPoC}^{⊤}X_{te}\left(e\right)\right]$.

The results reported here were computed using the SPoC implementation provided in the MNE toolbox by Gramfort et al. (2013a, 2014).

##### 3.1.2.1. Decoding Accuracy

In this specific scenario, the accuracy of the decoding provided by SPoC is assessed in terms of the linear correlation ρ between the estimated target labels $\hat{z}$ and the true labels ***z***.

#### 3.1.3. Convolutional Neural Network (ConvNet)

Finally, the third use-case for our *post-hoc* labeling framework shall be provided by a ConvNet as proposed and implemented by Schirrmeister et al. (2017). ConvNets provide an end-to-end decoding of raw EEG signals and thus may be a good method to chose when prior knowledge about relevant EEG features is missing. Specifically, we utilized a *shallow* ConvNet architecture. It focuses on both temporal and spatial convolutions and thus has the capacity to detect features in both time and spatial domains, similarly to features extracted by filters derived by CSP and SPoC. Unlike CSP and SPoC, however, the input representation of the EEG signals to the ConvNet does not assume any type of frequency pre-filtering. Instead, it consists of the *N*_*c*_ channels and *N*_*t*_ time points of the (epoched) raw EEG.

##### 3.1.3.1. ConvNet Architecture

**Figure 2:**
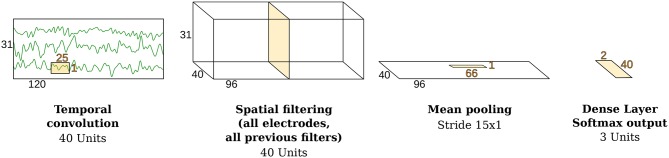
Architecture of the ConvNet. Modified from Schirrmeister et al. (2017) with the permission of the authors.

As shown in Figure 2, the temporal convolution step made use of a kernel size of 25 samples, containing 40 neural units. Subsequently, a layer with 40 units performed a spatial convolution step on all the channels. Finally, a log-power computation precedes a mean pooling stage and a fully connected layer with 3 units (softmax activation), one for each class.

##### 3.1.3.2. Decoding Accuracy

We chose classification accuracy as evaluation metric for the shallow ConvNet. Nevertheless, as in CSP and SPoC, any given metric might have been utilized for this purpose, depending on the focus of the analysis.

### 3.2. The EEG Dataset

#### 3.2.1. Signal Acquisition

The EEG signals for our use-case were recorded from seven healthy subjects (three females) with a mean age of 28 years. Seventy three minutes of EEG data on average were recorded in a single session while subjects sat calmly in front of a computer screen and performed the sequential visual isometric pinch task (SVIPT) (Reis et al., 2009). Given the paradigm-agnostic character of the *post-hoc* labeling framework, details about the SVIPT paradigm remain outside the scope of this paper but can be consulted in Meinel et al. (2016). EEG signals were recorded from 31 passive Ag/AgCl electrodes (EasyCap GmbH, Germany) placed according to the extended 10-20 system. Impedances were kept below 20 kΩ. All channels were referenced against the nose at recording time and were re-referenced against the EEG common average during the *post-hoc* analysis. The EEG signals were registered by BrainAmp DC amplifiers (Brain Products GmbH, Germany) at a sampling rate of 1 kHz, with an analog lowpass filter of 250 Hz applied before digitization.

#### 3.2.2. Pre-processing Only for *post-hoc* Labeling

All processing steps necessary to generate the *post-hoc* labeled datasets were performed in Matlab using the BBCI toolbox (Blankertz et al., 2016). The EEG signals were bandpass filtered between 0.2 and 48 Hz with a 5th order Butterworth filter and then sub-sampled to 120 Hz. Assuming the alpha band being in the focus of a benchmarking scenario, EEG data were further filtered with a 5th order Butterworth bandpass filter with cut-off frequencies at 8 and 12 Hz. This target frequency band can be modified according to different analysis goals, but for the sake of compactness in the use-case analysis, we have kept this parameter fixed. Finally, the label extraction procedure described in section 2.2.2 was applied, in order to obtain a labeled dataset comprised by ***X*** and ***z***.

For the generation of the datasets containing anatomical constraints on the sources, the publicly available *New York Head* (Huang et al., 2016) was used. It describes a finite element model containing 2,000 sources located on the cortical surface. These sources were subsampled from a highly detailed model containing 74,382 sources, which had been computed from a non-linear average of 152 human brains. The *New York Head* takes scalp, skull, cerebro-spinal fluid, gray matter, and white matter into account. Sources were assumed to be perpendicularly oriented wrt. the cortical surface, however, our framework could also be used with models that allow for free source orientation.

On the other hand, for the data-driven approach, a fixed number of 20 ICA components were extracted for each subject. Ideally, only components corresponding to actual neural sources should be selected for further analysis. For the identification of such neural components, the multiple artifact rejection algorithm (MARA) (Winkler et al., 2011) was applied, using a posterior probability threshold of 10^−8^ for components classified as having neural origin.

#### 3.2.3. Pre-processing for Algorithm Benchmarking

The pre-processing pipeline we chose to apply on the newly labeled data was selected to match the requirements of the decoding methods presented as use-cases; consequently, it is independent of the *post-hoc* dataset generation framework.

For outlier detection, the continuous EEG data ***X*** were bandpass filtered between 0.7 and 25Hz with a 5th order Butterworth filter. Segments of the continuous data with peak-to-peak amplitude exceeding 80 μV were marked as artifactual for later removal in the pre-processing pipeline. Only for CSP and SPoC, the original continuous data ***X***, was filtered by a 5th order Butterworth filter to the band of 8–12 Hz. For ConvNets, the original raw data was used. Then, EEG data and the target source ***z*** were segmented in non-overlapping windows of 1 s duration. At this point, epochs marked earlier as artifactual were removed. For the remaining segments, the epoch-wise average power of ***z*** was extracted and used as the target variable to train the decoding algorithms. For CSP and ConvNet, epoch-wise discrete labels were generated. For the binary classification tasks they were determined by the top and bottom 50th percentile, whereas the 33th and 66th percentiles limits defined the three-class problem. At this point we want to point out, that the epoching does not necessarily need to obey the original time structure of the experimental paradigm, under which ***X*** was recorded.

### 3.3. Sweep Over Three Dataset-Inherent Parameters

**Figure 3:**
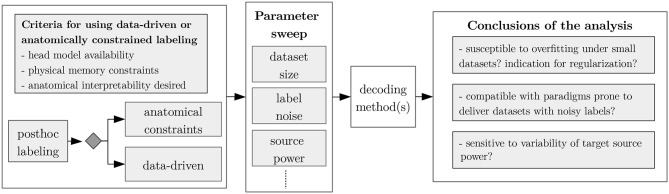
Typical use-case for the *post-hoc* labeling framework. Firstly, the kind of inverse mapping algorithm used shall be determined (e.g., ICA, MNE, or any other inverse mapping method). Afterwards, depending on the analysis goals, a subset of dataset parameters—as dataset size or label noise—can be selected for a parameter sweep, allowing to arrive to conclusions providing insight about the decoding methods evaluated.

The practitioner will probably strive for the best possible decoding performance. For doing so, he/she may have the choice between different decoding models or may try to improve upon existing methods. In a real application, the relative variance of the labels, dataset size, and label noise level are typically not or only weakly controllable, even though these dataset-inherent parameters may have a strong impact upon the decoding. In our benchmarking scenario, however, absolute control over these or similar dataset-inherent parameters is granted for free, thus allowing to investigate, under which conditions a decoding model is applicable or which aspects of an existing method should be improved in order to optimize the decoding performance. Along the lines of the illustrative chart shown in Figure 3, we provide three exemplary use cases, where we tested the robustness of the three decoding methods wrt. the aforementioned parameters, namely: (1) relative variance of the labels, (2) dataset size, and (3) label noise level.

#### 3.3.1. Relative Variance of the Labels

First, machine learning methods favor datasets that contain high contrast in the labels. For example, Meinel et al. (2018) demonstrated that SPoC decoding performance is positively correlated with the variance of the target labels ***z*** over epochs *e*. In our current contribution, we analyzed the performance of the decoding methods for three type of target sources: sources with high, medium, and low power variability, each corresponding to the respective subject-wise *tertile membership* of the source power variability (***z***-variance). Note that when using ICA for generating $S_{d}$, the scaling of the sources ***S*** is unknown. This does not represent any drawback for our framework; however, to allow for the analysis of performance with respect to ***z***-variance, each of the sources in $S\in S_{d}$ were normalized wrt. their ℓ_2_-norm and scaled using the average covariance of each source with all the channels, i.e., ${\tilde{s}}_{i}=\left(1/N_{c}\underset{N_{c}}{\sum }s_{i}x_{i}^{\text{T}}\right)s_{i}$; with ${\tilde{s}}_{i}$ being the *i*-th row in $\tilde{S}\in S_{d}$, from which the ***z*** labels are finally extracted, according to section 2.2.2.

#### 3.3.2. Dataset Size

Second, each decoding model's sensitivity wrt. the *number of training epochs* was evaluated by sweeping from 50 to 2,000 epochs, as larger datasets prevent overfitting and deliver more robust models.

#### 3.3.3. Label Noise

Third, the influence of *label noise* was investigated, which was either imposed upon ***z*** (in the case of continuous labels in regression problems) or upon ***y*** (for classification problems). For both types of problems, the intensity of the noise was defined, respectively, as $\left\{\xi_{n}^{reg},\xi_{n}^{class}\right\}\in \mathbb{R},\;0<\xi_{n}^{reg}<1,\;0<\xi_{n}^{class}<1$, specifically:

*Label noise for regression:* The variable $\xi_{n}^{reg}$ controls the correlation ρ_*n*_ between the original clean labels ***z*** and more challenging labels ***z***_*n*_, such that $\xi_{n}^{reg}=\left(1-\rho_{n}\right)$ holds. Subsequently, noisy labels ***z***_*n*_ are defined as

(9) $$\begin{array}{l} z_{n}=z+\frac{1-{\left(1-\xi_{n}^{reg}\right)}^{2}}{{\left(1-\xi_{n}^{reg}\right)}^{2}}\text{var}\left(z\right)\eta \end{array}$$

where **η** is a normally distributed random variable.

*Label noise for classification:* The variable $\xi_{n}^{class}$ for discrete labels is defined as

(10) $$\begin{array}{l} \xi_{n}^{class}=2\left(1-P\left[y_{n}(e)=y(e)|y(e))\right]\right) \end{array}$$

that is, $\xi_{n}^{class}$ controls the probability of assigning a given epoch *e* to a class different from the ground truth. For multiclass problems, if *y*_*n*_(*e*)≠*y*(*e*), then *y*_*n*_(*e*) is assigned with equal probability to any of the remaining classes.

Sweeping over the three hyperparameters delivered more than 10,000 evaluations of different dataset configurations. The performance of each configuration is assessed by a chronological 5-fold cross-validation procedure. All results shown are based on the data of all the seven subjects. The results shown for SPoC and CSP were computed using the dataset generation framework based on data-driven source constraints, whereas for ConvNet, the version with physiologically constrained sources was used.

## 4. Results of Use-Cases

**Figure 4:**
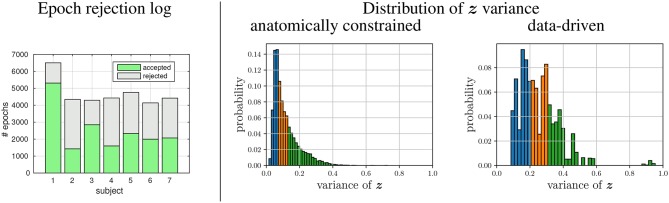
Characteristics of the generated datasets. **(Left)** accepted and rejected (artifactual) 1-s epochs for each of the subjects. **(Right)** Pooled over all sources derived from the seven subjects, the distribution of the variance of labels ***z*** is given, as observed during the data/label generation using either the head model or the data-driven approach. Color encode the tertile membership of the ***z***-variance: 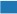 low varying labels, 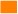 medium varying labels, and 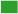 high varying labels.

The datasets generated using the *post-hoc* labeling framework are characterized by Figure 4. Per subject, an average of 4,700 epochs could be obtained from only 73 min of average EEG recording time, while an average of 2,136 epochs were rejected as artifactual. Thus the proposed framework yields an acceptance rate of approximately 55%. The accepted epochs could then be labeled in multiple ways, as each source can be utilized to define a label set. Per source, the resulting labels were analyzed for variance. We found, that the distribution of all label variances approximates a gamma distribution (see histograms in Figure 4), both for the labels extracted via MNE and those extracted by the data-driven fastICA approach.

**Figure 5:**
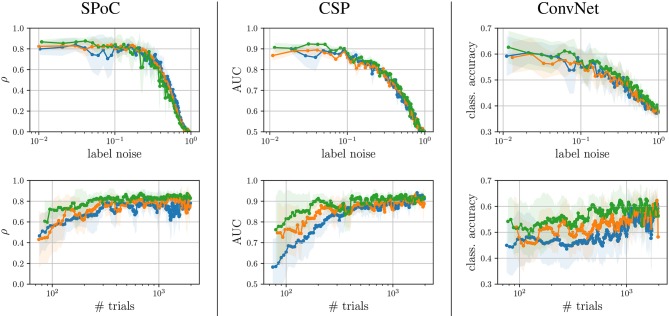
Performance analysis wrt. to label noise and dataset size, discriminated for each tertile membership of ***z***-variance, i.e., 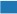 first tertile, 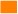 second tertile, and 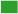 third tertile. (The corresponding shaded areas indicate one standard deviation).

**Figure 6:**
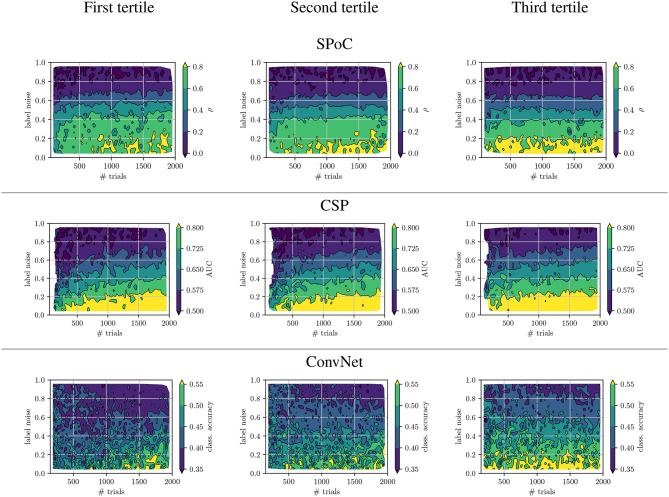
Biparametrical analysis of performance wrt. to tertile membership of the target variable ***z*** variance.

Next, we exemplify how the sensitivity of the three use-case algorithms SPoC, CSP, and ConvNet toward dataset-inherent parameters can be analyzed using the proposed framework. The top row of Figure 5 shows the performance metrics obtained by a sweep over the parameter *label noise* while maintaining the full dataset size. The bottom row depicts the influence of the *dataset size* upon the performance, while no label noise was applied ($\xi_{n}^{class}=\xi_{n}^{reg}=0$). For each subplot, results have been grouped into tertiles defined by ***z***-variance. In addition, Figure 6 provides a full bi-parametric analysis separately for the ***z***-variance tertiles.

We observed, that label noise values of $\xi_{n}^{reg}>0.2$ significantly reduced SPoC's performance score ρ. Interestingly, the variance of the labels ***z*** does not seem to play a systematic role for SPoC's decoding quality, if the training set is large. However, SPoC's performance suffers a drop for dataset sizes of ≈ 300 epochs or less, and this drop is most pronounced for the low and medium groups of ***z***-variance. It can also be observed that high label noise cannot be compensated for by SPoC, even with large training datasets. However, for limited amount of noise, a larger dataset may be a sufficient countermeasure, which can be obtained by adapting the experimental design of a given paradigm in order to improve the data collection efficiency. The method CSP shows a similar behavior to SPoC. The critical thresholds of the investigated parameters, for which the performance dramatically drops, are around a label noise value of ξ_*n*_ ≈ 0.1 and a dataset containing ≈400 samples. These results might be an indication for applying regularization techniques, specially if CSP or SPoC are to deal with small datasets. This conclusion agrees with state-of-the-art studies, e.g., by Meinel et al. (2018).

ConvNet models show a high performance variance over the full spectrum of both parameters. Its performance scores are very sensitivity wrt. the number of epochs and label noise: this data-hungry method requires approx. 1,000 epochs under no label noise to reach peak performance. Even small amounts of label noise influence ConvNet's performance, with label noise larger than $\xi_{n}^{class}\approx 0.1$ leading to a pronounced decline of the network's performance. It is necessary to remark that the 3-class classification task solved by ConvNet is the most complex one of the use-cases analyzed. This may partially explain the increased sensitivity of the method to the dataset size and label noise. However, another likely reason for such sensitivity is the large number of free parameters of the network to be tuned during its training, indicating that the complexity of the network should be reduced if the dataset size is not large enough.

## 5. Discussion

For the development of decoding algorithms, an ideal testbench should be capable of providing large amounts of data, clean labels, and realistic neural dynamics. Unfortunately, state-of-the-art approaches lack one or several of these properties. To address this, we have introduced a labeled dataset generation framework. Its key idea is to implement a *post-hoc labeling* of (potentially very long) paradigm-agnostic pre-recorded M/EEG signals.

### 5.1. Advantages Over State-of-the-Art Frameworks

The *post-hoc* labeling framework allows to generate relatively large labeled datasets based on real neural signals and by doing so, it prescinds from making critical assumptions about neural dynamics. As a clear advantage of the proposed framework, the labels can be deterministically recoverable from the available data, thus, they are provided free of noise, from the perspective of the decoding methods. Furthermore, the *post-hoc* labeling of paradigm-agnostic M/EEG recordings offers greater efficiency in terms of data use. Compared to real datasets whose labels depend on the paradigm they were recorded under, our *post-hoc* labeling can also make use of recorded idle periods or preparatory intervals. For the provided EEG dataset, this led to an exploitation of effectively 55% of the overall M/EEG recording time for training and test data generation.

Our framework allows for absolute control over important parameters of the generated dataset and provides full knowledge about the statistical and—in case of the head model—anatomical properties of the target sources. It provides an ideal starting point for comparing competing decoding methods, as it yields insight into the data conditions under which methods stand out among the competing others.

Nevertheless, one relevant parameter unfortunately remains outside the control of our framework: the amount of sensor noise. This parameter is determined by the available real-M/EEG signals and can not be improved (only worsened) *post-hoc*. Here, synthetic data generation approaches have a theoretical advantage, as they can control the level of sensor noise. In practice, however, it may not be straightforward to determine noise levels during synthetic data generation in order to match real experimental conditions.

### 5.2. Application in Development of Neural Decoding Methods

With the three use-cases presented as exemplary analyses enabled by *post-hoc* labeling, we intent to show how our framework can be used to investigate strengths and limitations of arbitrary decoding algorithms under different scenarios. For example, it could be easily observed, that the performance differences among labels with different variability is marginal, if the number of epochs surpasses a critical, method-specific threshold. However, for datasets below this threshold, the strongest varying labels showed a better performance than those belonging to the first and second tertile of label variability. Using the *post-hoc* labeling framework was an effective way to investigate these thresholds as well as the influence of label noise. However, not only the required (minimum) amount of training data or feasible levels of label noise can be examined with the framework, also other specific constraints could easily be incorporated during the generation of the benchmark datasets. Examples are prior knowledge about central frequency, strength, or anatomical location of sources.

Nevertheless, frameworks using purely synthetically generated signals or real EEG recordings with paradigm specific labels have their place, next to the *post-hoc* labeling framework, in the development pipeline of neural decoding methods. Early development stages of decoding algorithms may benefit from strictly controlled simulation environments, as shown for example in the recent work presented by Krol et al. (2018). Furthermore, in late development stages and prior to deployment in real-world applications, validation on strictly real scenarios is necessary, for example, using the benchmark framework provided by Jayaram and Barachant (2018).

### 5.3. Considerations About Non-stationary Dynamics

By using MNE and ICA for inverse mapping, as shown in the exemplary use-cases, we assume a stationary mapping between M/EEG signals and neural sources, which may not always be true, specially for long recordings. Such assumption should be kept in the foreground, mainly in two scenarios: First, when claiming that the generated target sources exclusively correspond to a particular source with specific anatomical or physiological interpretation, since a stationary inverse mapping under non-stationary conditions would potentially deliver a time-varying mixture of underlying neural sources. Second, when benchmarking neural decoding methods designed to deal with non-stationary dynamics, since *post-hoc* labeling using stationarity assumptions may deliver a dataset where the strengths of any adaptive decoding method cannot be properly evaluated.

Using ICA and MNE in our use-cases was motivated by the observation, that the most popular decoding methods (for example, CSP, SPoC, or convNets) predominantly assume an underlying within-session stationary process. Challenging those popular decoding methods with labels derived by, e.g., STOUT or adaptive ICA would probably have an impact upon these decoding methods comparable to label noise.

Fortunately, the flexibility provided by the *post-hoc* labeling framework facilitate the use of inverse mapping methods, capable of dealing with underlying non-stationary processes. For example, state-of-the-art source reconstruction methods as TF-MxNE (Gramfort et al., 2013b) or STOUT (Castaño-Candamil et al., 2015a) are designed to extract neural sources from non-stationary M/EEG recordings, and could be employed in our *post-hoc* labeling framework, instead of MNE. Likewise, adaptive blind-source separation methods (usually deployed in online scenarios) can be used to perform a data-driven inverse mapping without assuming stationary dynamics (Hsu et al., 2015), as an alternative to the ICA procedure presented. Finally, under the assumption of piecewise stationarity, long recordings may be segmented into locally stationary windows—e.g., using statistical features of the spectrogram of the signals (Hory et al., 2002)—, and then the proposed *post-hoc* labeling framework can be carried out in the resulting (stationary) epochs.

To wrap up, Table 1 summarizes the properties of our contribution (*post-hoc labeled data*) compared to that of other testbench approaches.

**Table 1:** Comparison of advantages and disadvantages of two state-of-the-art testbench scenarios against the proposed novel *post-hoc labeling* framework.

**Dataset type**	**Dataset size**	**Label noise ctrl**.	**Sensor noise ctrl**.	**Real statistics and dynamics**
Synthetic	Large 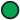	Yes 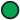	Yes 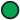	No 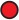
Real EEG	Small 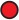	No 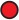	No 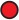	Yes 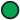
*Post-hoc labeling*	Medium/large 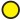 / 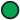	Yes 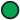	No 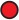	Yes 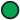

To facilitate the adoption of the *post-hoc* labeling framework as a tool for developing and testing decoding algorithms for oscillatory neural phenomena, both the source code and datasets utilized in the use-case scenarios have been made publicly available^1^ (Castaño-Candamil et al., 2017a).

## Data Availability

All datasets generated for this study are included in the manuscript and/or the supplementary files.

## Author Contributions

SC-C, AM, and MT: conceived the methods, collected dataset, and contributed analysis tools. SC-C and AM: method implementation. SC-C: performed the experiments. SC-C and MT: wrote the paper.

### Conflict of Interest Statement

The authors declare that the research was conducted in the absence of any commercial or financial relationships that could be construed as a potential conflict of interest.

## Footnotes

^1^https://github.com/bsdlab/Post-HocLabeling
